# Whole genome sequencing of marine organisms by Oxford Nanopore Technologies: Assessment and optimization of HMW‐DNA extraction protocols

**DOI:** 10.1002/ece3.8447

**Published:** 2021-12-07

**Authors:** Sonia Boughattas, Dana Albatesh, Albandari Al‐Khater, Bruno W. Giraldes, Asma A. Althani, Fatiha M. Benslimane

**Affiliations:** ^1^ Biomedical Research Center Qatar University Doha Qatar; ^2^ Barzan Holdings Doha Qatar; ^3^ Environmental Science Center Qatar University Doha Qatar

**Keywords:** DNA extraction, marine species, ONT sequencing, protocol optimization, whole genome sequencing

## Abstract

Marine habitats are Earth's largest aquatic ecosystems, yet little is known about marine organism's genomes. Molecular studies can unravel their genetics print, thus shedding light on specie's adaptation and speciation with precise authentication. However, extracting high molecular weight DNA from marine organisms and subsequent DNA library preparation for whole genome sequencing is challenging. The challenges can be explained by excessive metabolites secretion that co‐precipitates with DNA and barricades their sequencing. In this work, we sought to resolve this issue by describing an optimized isolation method and comparing its performance with the most commonly reported protocols or commercial kits: SDS/phenol–chloroform method, Qiagen Genomic Tips kit, Qiagen DNeasy Plant mini kit, a modified protocol of Qiagen DNeasy Plant kit, Qiagen DNeasy Blood and Tissue kit, and Qiagen Qiamp DNA Stool mini kit. Our method proved to work significantly better for different marine species regardless of their shape, consistency, and sample preservation, improving Oxford Nanopore Technologies sequencing yield by 39 folds for *Spirobranchus* sp. and enabling generation of almost 10 GB data per flow cell/run for *Chrysaora* sp. and *Palaemon* sp. samples.

## Graphical abstract

Optimum ONT sequencing runs observed with the bespoke protocol.

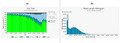

## 1 INTRODUCTION

Previous biogeographic studies revealed exclusive divergence of animal species composition in Gulf region, particularly in Qatar, as its marine ecosystems are driven mainly by high temperature and salinity with a semi‐closed basin, depending thus on water input from the Indian Ocean, supplied on the Gulf's southeast coast, and creating a marine barrier for different species as, for example, Decapods (Al‐Khayat & Giraldes, 2020). Due to these peculiar conditions, the fauna and flora have adapted to survive under extreme conditions (Al‐Khayat, 2010), contributing to the evolution of the marine species in this area and consequently enriching the environment with endemic species (Fotedar et al., 2019; Giraldes et al., 2020; Kardousha et al., 2016). The identification of some of these species has so far been based on conventional taxonomic parameters, although this type of analysis can be notoriously difficult (Bork, 2015). The morphological convergence of marine organisms and their phenotypic plasticity often leads to imprecise taxonomic assignment. Studying their genomes can clarify the phylogenetic relationships among and within genera and species (Panova et al., 2016). However, the molecular approach has been impeded by scarcely available genomic data.

We recently, launched a project that aims to sequence whole genomes of Qatar's marine species to identify potential new marine organisms and create a library for Qatar's marine species. We applied Oxford Nanopore Technologies (ONT) platforms to achieve this aim as it generates long reads that facilitate genome assemblies. The ONT’s sequencing flow cells utilize hundreds of nanopores concurrently for reading single‐stranded DNA at up to 450 nucleotides per second, resulting in several gigabases of sequence during a 2‐day run. As the technology does not rely on PCR or discrete strand synthesis events, DNA fragments can be arbitrarily long (Jansen et al., 2017). However, to successfully utilize this genomic approach, obtaining a pure and high molecular weight (HMW) DNA is required. Such DNA extraction from marine organisms is yet an arduous task due to the various tissue consistency and high polysaccharide, polyphenol, and other secondary metabolites content co‐extracted with the DNA (Ramakrishnan et al., 2017). These contaminants can inhibit the activity of the enzymes during library preparation, rendering the DNA useless for downstream applications.

Therefore, we surveyed a wide range of homogenization methods and genomic DNA extraction protocols either using chemical extraction (Lin et al., 2016) or commercial extraction kits to assess their efficiency in providing high‐quality HMW‐DNA that is suitable for long reads sequencing.

## 2 MATERIALS AND METHODS

Specimens of the intertidal belt‐forming worm *Spirobranchus* sp. were collected along Qatari coasts. Most specimens were found in dense aggregations containing thousands of individuals covering the rocks’ surfaces. They were thus removed from their tubes by carefully cracking the tubes with a knife, placed in vials, and transported to the laboratory for further analysis (Pazoki et al., 2020).

Due to their small size, two worm specimens (~70 mg) were used for DNA isolation for each of the six investigated extraction protocols: Sodium dodecyl sulfate (SDS)‐phenol/chloroform protocol (50 mM Tris‐HCl pH 8, 0.1 M EDTA, 1% SDS, 0.2 M NaCl, and 100 μg/ml proteinase K) as reported previously (Lin et al., 2016) and the Qiagen Genomic Tips 100/G kit as recommended by ONT (Jansen et al., 2017) with a liquid nitrogen homogenization step. Four other protocols were also investigated based on three wildly used commercial kits when dealing with difficult samples (DNeasy Blood & Tissue kits; QIAamp DNA Stool mini kit; Qiagen's DNeasy Plant mini kit; and modified DNeasy Plant protocol). The addition of a mechanical homogenization step was also assessed; and DNA yield, purity, and sequencing profile were compared (Angthong et al., 2020). The first three protocols were carried out as per manufacturer's guidelines. For the fourth protocol that utilizes the DNeasy Plant mini kit, a proteinase digestion step was introduced (50 µl of 20 mg/ml stock solution) during the sample lysis (Table 1).

**TABLE 1:** Assessment of the different extraction protocols

	Chemical	Genomic tips LN©	Genomic tips M©	DNeasy plant kit©	DNeasy plant kit modified	DNeasy tissue kit©	QIAamp DNA stool kit©	Optimized protocol
Homogenization	Liquid nitrogen	Liquid nitrogen	Mechanic	Mechanic	Mechanic	Mechanic	Mechanic	Mechanic
Lysis buffer	SDS buffer	G2	G2	AP1	AP1	ATL	ASL	G2
Protease treatment	Yes	Yes	Yes	No	Yes	Yes	Yes	Yes
Lysis incubation	2 H	2 H	2 H	10 min	10 min	Overnight	15 min	Overnight
Inhibitor's removal	No	No	No	No	No	No	Inhibitex	Inhibitex
Extraction interface	Phenol/chloroform	Resin column	Resin column	Silica column	Silica column	Silica column	Silica column	Resin column
A260/A280	2.35	1.98	1.95	1.44	1.67	1.76	1.9	1.83
DNA yield ng/μl	4.12	4.42	6.48	41.4	20.8	14.3	11.2	15.9

Note

LN with Liquid Nitrogen lysis step; M with Mechanical lysis step; ©: extraction protocol according to the manufacture guidelines.

A new optimized protocol concocted from the combination of different steps of the common methods was also investigated. Initial wash steps were performed three times by PBS buffer for 5 min at 4000 × *g*. Mechanical homogenization was then achieved in Lysis Buffer G2, provided with the Genomic Tips 100/G kit, using the TissueRuptor II (Qiagen) on ice at low speed 2 for 10 s (a second step may be needed in case of specimens with hard shells). Proteinase K (20 mg/ml) and RNAse A (200 µg/ml) were added as per the manufacturer's guidance and incubated at 50°C. Different incubation periods were tested: incubation for 2 h as recommended and 16 h (overnight) incubation. Then, a centrifugation step was performed for 5 min at 4000 × *g* to pellet the debris. Two tablets of InhibitEX (Qiagen) were added to the obtained supernatant and solubilized by inversion followed by 5 min incubation at room temperature. Another centrifugation step was performed for 5 min at 4000 × *g* and again the pellet was discarded. The retained supernatant was then subjected to extraction by gravity flow procedure according to the Genomic Tips kit guidelines with some adjustments: an extra‐incubation step of 5 min at room temperature after the addition of the isopropanol and another extra‐incubation step of 1 h at 37°C for the final elution with pre‐warmed eluting buffer.

The extracted DNA was subjected to quantification using a Qubit dsDNA High Sensitivity Assay kit on the Qubit^®^ 4.0 Fluorometer (ThermoFisher Scientific). Purity and fragment length were checked using the Nanodrop ratio A260/A280 and a 0.7% agarose gel visualization, respectively (Ketchum et al., 2018). Quality was also evaluated by PCR amplification using two pairs of primers targeting nuclear and mitochondrial genes and amplifying two different sizes of DNA fragments (Halt et al., 2009).

Extracted DNA was then subjected to library preparation using ONT’s Ligation Kit (SQK‐LSK109) according to the manufacturer's guidelines. Briefly, genomic DNA (800 ng) was subjected to end repair and a tailing by NEBNext FFPE DNA Repair mix and NEBNext Ultra II End repair/dA‐tailing modules (New England Biolabs) prior to the ligation. The sequencing adaptors were ligated using NEBNext Quick Ligation module (New England Biolabs) after an AMPure XP (Beckman Coulter, California, USA) magnetic beads purification step. After a final product clean‐up using the Long Fragment Buffer (LFB), the sequencing library was loaded to a primed FLO‐MIN106 flow cell on a GridIon device for a 48‐h run. The sequencing device control, data acquisition, and real‐time basecalling were carried out by the MinKNOW software. Translocation speed, pore occupancy, cumulative output, and N50 of the read length were continuously checked during the sequencing runs.

## 3 RESULTS

**FIGURE 1:**
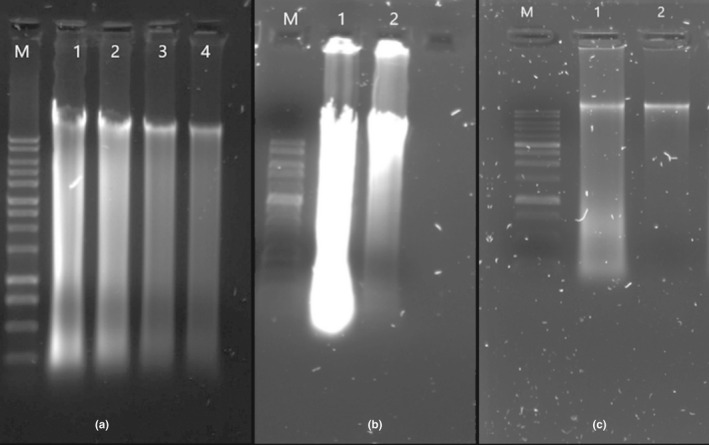
Agarose gel 0.7% electrophoresis representing the DNA extraction yield of the different tested protocols with M: 1Kb ladder. Five microliters of each DNA product were loaded on the gel. (a) 1. DNA extract of phenol–chloroform protocol, 2. DNA extract of Genomic Tips kit, 3. DNA extract of Tissue kit, and 4. DNA extract of Stool kit. (b) 1. DNA extract of plant kit protocol; 2. DNA extract of modified plant kit protocol. (c) DNA extracts from optimized protocol: 1. DNA extract with 2 h lysis step incubation; 2. DNA extract with overnight lysis step incubation

Using the most commonly reported approaches: SDS‐Phenol/chloroform protocol and the Genomic Tips protocol with liquid nitrogen snap freezing as a pre‐treatment step, the purity of the retained DNA was 2.35 and 1.98, respectively, as determined by the A260/A280 ratio (Table 1). Signs of DNA degradation/contamination were observed after agarose gel electrophoresis (Figure 1a).

**FIGURE 2:**
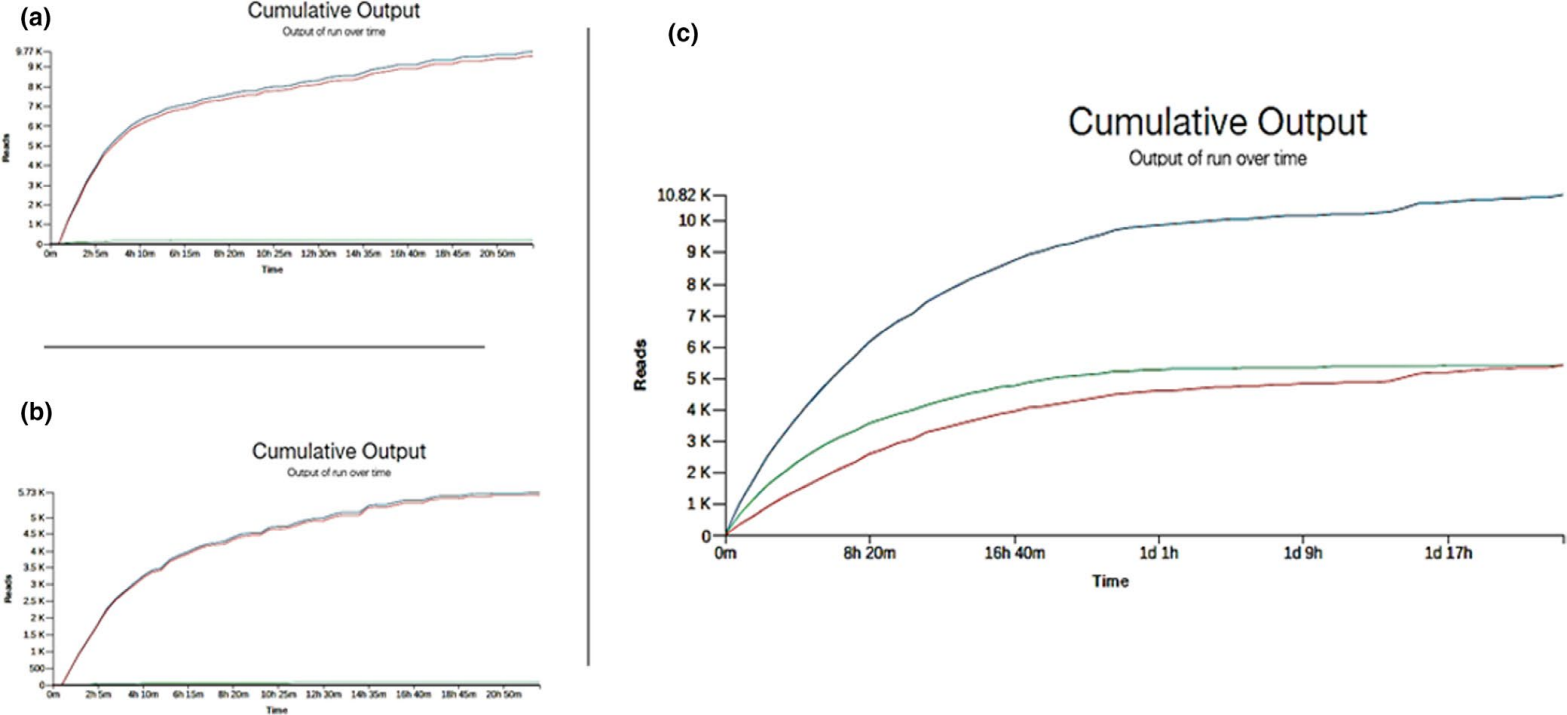
Graphical representations of the cumulative output of ONT runs over time. *X* axis: Run time; *Y* axis: Reads output. Blue color summarizes the cumulative reads of the run, Red color line indicates the failed reads, and Green color line indicates the passed reads. (a) The SDS‐Phenol/Chloroform protocol; (b) Genomic Tips kit protocol; and (c) Modified‐Plant kit protocol

When the obtained DNA was subjected to ONT sequencing, the translocation speed was outside the recommended range right from the beginning of the sequencing run. For both protocols, pore occupancy did not exceed 40%, and more than 98% of the sequencing reads were classified as failed. According to the default setting of ONT runs, the reads are classified as failed after MinKNOW basecalling runs through a qscore filtering process. This qscore filtering only looks into the quality of the data and does not consider minimum read length, consequently any reads with qscore value lower than 7 are sent to the “fail” folder while basecalling. The obtained passed reads are represented by a flat green line almost confounded with the *X* axis (Figure 2a,b). DNA extractions and ONT sequencing runs were repeated more than once; the same observations of DNA degradation and failed ONT reads were obtained.

To minimize any bench‐to‐bench variability, both extracts were also subjected to automatic library preparation by VolTRAX V2 device using the VolTRAX Sequencing kit (VSK‐VSK002) and the VolTRAX Configuration kit (VCK‐V2002). Library preparations were unsuccessful as the device was not able to complete the process due to blockage occurrence. Same observations were recorded even after the experiment was repeated twice with diluted DNA input.

Using the TissueRuptor II for the homogenization step and silica membrane‐based kits, the recorded DNA purity A260/280 ratio was 1.44, 1.67, 1.76, and 1.9 for the Plant, modified‐Plant, Tissue, and Stool Qiagen extraction protocols, respectively (Table 1).

Both Plant kit‐based protocols yielded higher DNA quantity but with consequent contaminants presence even if the modified‐Plant protocol extract showed less smearing on the agarose gel than the original Plant extraction protocol extract (Figure 1b).

When sequencing the DNA extracted by the modified‐Plant protocol, successful runs are observed, with pore occupancy reaching 53%. However, almost half of the 10.82 K obtained reads did not pass the minimum required quality and were classified as failed (Figure 2c, Table 2).

**TABLE 2:** ONT performance comparison of different extraction protocols

	Chemical	Genomic tips LN	Genomic tip M	DNeasy plant kit modified^a^	DNeasy tissue kit	QIAamp DNA stool kit	Optimized protocol		
	*Spirobranchus* sp. (Worm)						Worm	Shrimp	Jellyfish
Starting pore occupancy (%)	31	38	49	53	27	32	38	82	52
Total reads	5.73 kb	9.77 kb	27.06 kb	10.82 kb	392.74 kb	46.45 kb	1.74 Mb	4.5 Mb	1.36 Mb
Passed reads (%)	<2	<2	<4	50	84	86	93	89	81
Failed reads (%)	>98	>98	>96	50	16	14	7	11	19
N50	1.9 kb	2.05 kb	2.18 kb	9.23 kb	1.56 kb	3.91 kb	13.82 kb	7.44 kb	24.03 kb
Estimated bases	6.96 Mb	10.21 Mb	27.94 Mb	36.22 Mb	326.27 Mb	115.43 Mb	3.46 GB	9.95 GB	8.64 GB

^a^ As the DNA product of DNeasy Plant kit represents a consequent smear, it was not run for ONT sequencing. Only the DNA extracted by DNeasy Plant kit Modified protocol was sequenced.

Successful sequencing runs with a majority of called passed reads were obtained with the extracts of the Tissue and Stool kits. The Stool kit extraction protocol showed increased output with higher N50 read length: 3.91 kb compared to 1.56 kb observed by the Tissue extraction protocol (Table 2).

**FIGURE 3:**
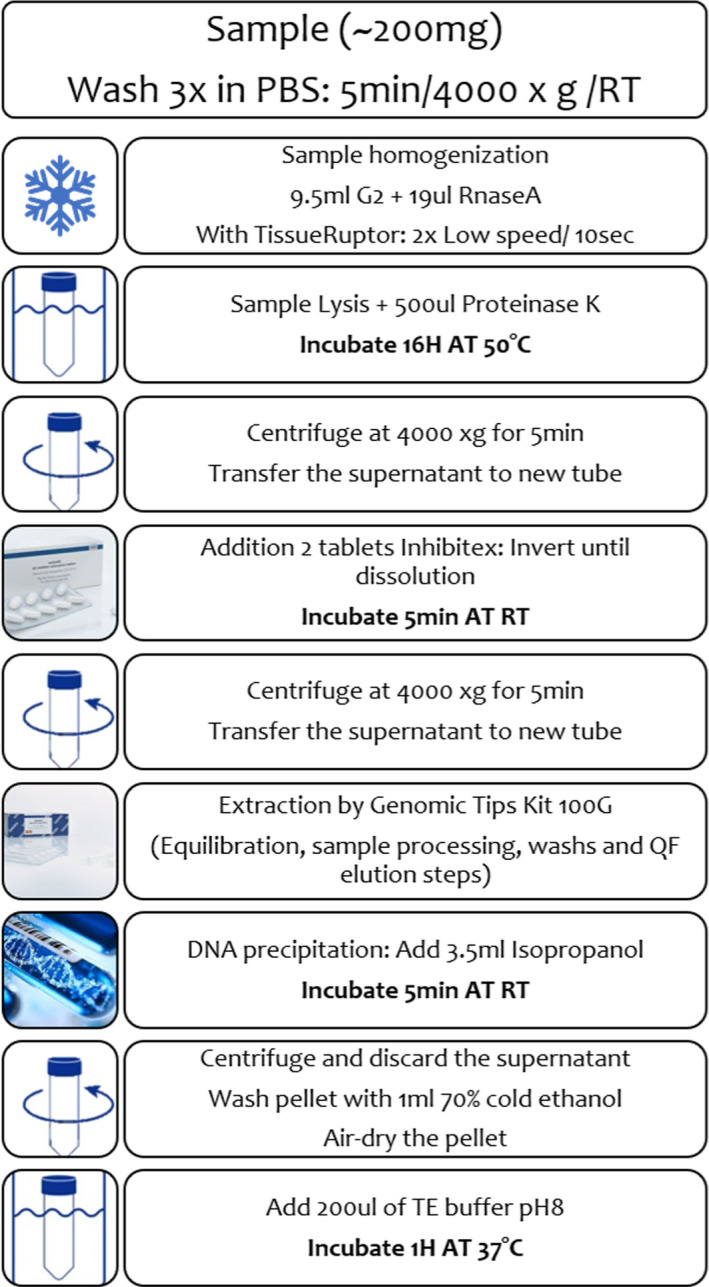
Flowchart representation of step‐by‐step optimized protocol

For the optimized protocol (Figure 3), the tissue lysis, the incubation conditions of the proteinase K, the DNA precipitation, and the elution steps were optimized. When the lysis step was carried out as recommended in commercial kits (2 h), a substantial smear on the agarose gel was detected. An increased incubation time to overnight resulted in a more efficient lysis with a clear genomic DNA band and minimal smear (Figure 1c). According to the Genomic Tips manufacturer's guidelines, 1–2 h incubation at 55°C is required for the final elution step. However, evaporation of the elution buffer volume was recorded when proceeding with our samples (decreased elution volume). The modification to 1 h incubation at 37°C gave satisfactory results. The yield of DNA increased also when we added a 5 min incubation step after the addition of the precipitating chemical (isopropanol) (Figure 3). Optimal nucleic purity was recorded as evident by A260/A280 ratio of 1.83 (Table 1), which suggests that the DNA extraction was free of proteins and polyphenolics/polysaccharide compounds. Sequencing of the extract resulted in a read length N50 of =13.82 kb with an increase in the ONT data generation by 39‐fold comparing to the mean of all the previous tested protocols (Table 2).

**FIGURE 4:**
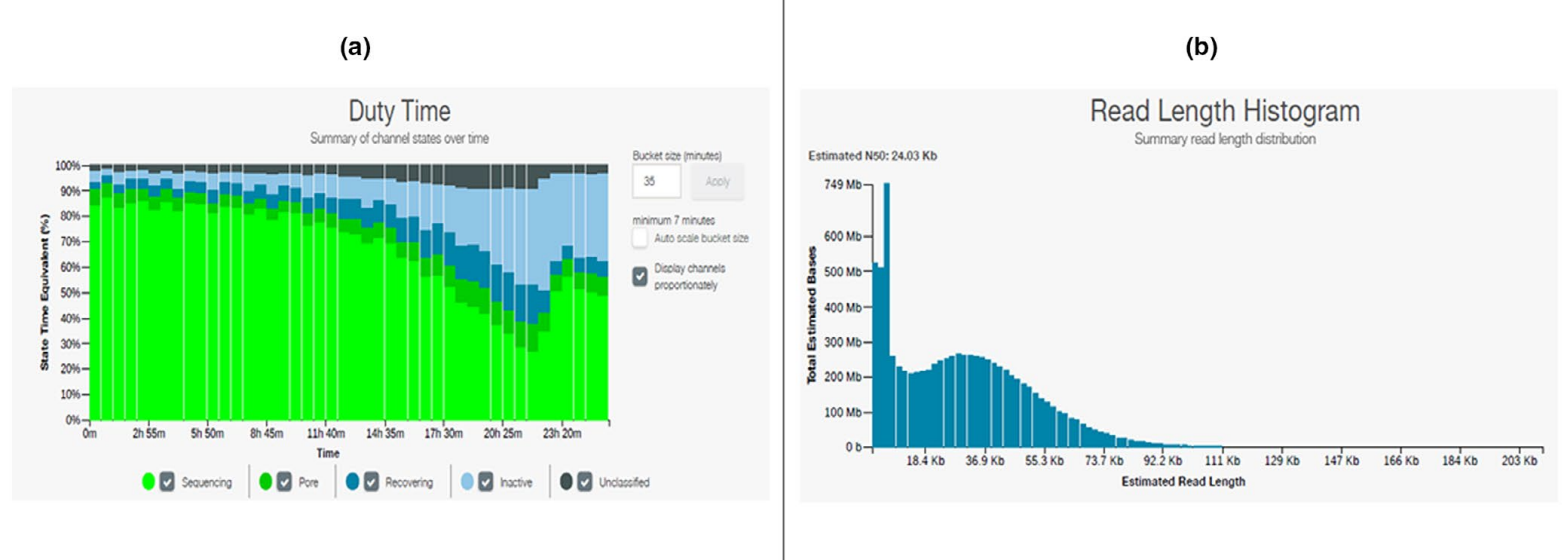
Graphical representation of optimized ONT runs. (a) Flow cell occupancy (Bright green/sequencing pores) with *Palaemon* sp. DNA. (b) Summary of the read length distribution observed with *Chrysaora* sp. DNA

When applying the same protocol for two other marine species (up to ~200 mg of tissue) with different tissue consistency: jellyfish sample (*Chrysaora* sp.) and shrimp sample (*Palaemon* sp.), the A260/A280 ratio ranged from 1.85 to 1.89, respectively (Table 1). When sequenced, flow cell occupancy of more than 82% was recorded (Figure 4a) as well as 9.95 GB of generated data for *Palaemon* sp., whereas sequencing of *Chrysaora* sp. generated 8.64 GB of data with a read length N50 of =24.03 kb (Figure 4b) within a 24‐h run (Table 2).

**FIGURE 5:**
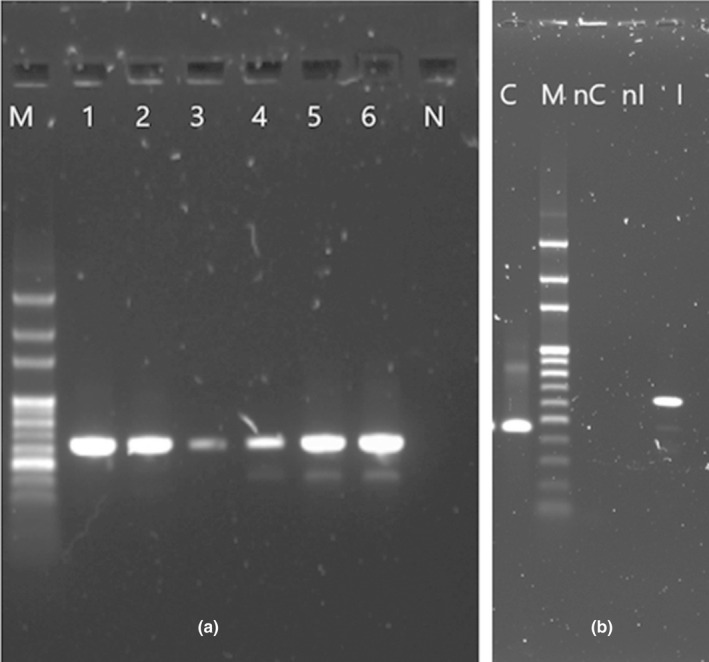
Agarose gel electrophoresis 1.2% of PCR products from different DNA extracts. Three microliters of each DNA product was loaded on the gel. (a) (ITS gene amplification): M 100 bp ladder; 1. stool extract; 2. tissues extract; 3. phenol/chloroform extract; 4. genomic tips extract; 5. plant extract; 6. modified plant extract; N negative control. (b) C optimized extract amplified by Cytb gene; M 100 bp ladder; nC negative control of Cytb gene; nI negative control of ITS gene; I bespoke extract amplified by ITS gene

It is noteworthy that the quality of DNA (A260/280, agaroses gel) was not always indicative of its amplification capabilities as all the DNA extracts from the seven protocols were amplified successfully for two independent genetic markers (Figure 5).

## 4 DISCUSSION

Investigating the marine organism's genomes can bring interesting insights into clarifying phylogenetic relationships among and within different genera and species, thus shedding light on some plasticity and adaptability characteristics of the species. However, a lack of genomic data from marine fauna is noted, which may be limited by the fastidious high‐quality DNA extraction in sufficient amounts (Chakraborty et al., 2020). Marine organisms naturally retain composites that interfere not only with the extraction of high molecular weight DNA but also with downstream molecular analysis. These inhibitory composites include polysaccharides, polyphenolics, pigments, chitin, and other secondary metabolites, typically secreted within tissues of marine organisms and thus observed in high concentrations (Bork, 2015). Consequently, DNA extraction is quite challenging as the polysaccharides intensify the viscosity of the extraction buffers, thus, impeding agitation/pipetting and inhibiting the activity of DNA enzymes. In addition, the polyphenol oxidation obstructs the DNA pellet solubilization and potentially inhibits any further downstream DNA library preparations (Maeda et al., 2013).

Our objective in this analysis was to survey how DNA extraction approaches vary and influence Oxford nanopores sequencing output of marine species with different consistency (Ketchum et al., 2018) and thus, optimize the conditions for successful sequencing runs.

For non‐destructive DNA extraction methods, a homogenization of the target sample in liquid nitrogen is recommended prior to the extraction procedure. We used this approach for the two most commonly reported extraction protocols. The first widely reported protocol within the marine species extraction uses phenol and chloroform with SDS buffer to extract the DNA from cellular molecules and debris (Ferrara et al., 2006; Pinto et al., 2000; Sokolov, 2000). We utilized this protocol to extract DNA from a worm that is habitant in the Arabian Gulf near the cost of Qatar. As *Spirobranchus* sp. was the organism with the smallest genome within the collection, we used it as a start model. However, the DNA extracted using these chemicals was not successfully sequenced on the ONT GridION platform.

The second DNA extraction protocol followed the guidelines of the Genomic Tips kit as recommended by the ONT experts (Jansen et al., 2017). According to the manufacturer, the kit operates by gravity flow and thus provides less DNA damage that might be observed with the centrifugation steps. The kit also uses unique anion‐exchange technology to purify high molecular weight DNA up to 150 kb with an average length of 50–100 kb. DNA extracted using this kit with a liquid nitrogen solubilization step also failed to sequence well the GridION platform. This led to the notion that the common liquid nitrogen homogenization step may be questionable, particularly when improvements were noticed after it was replaced with a mechanical homogenization within the Genomic Tips kit extraction (Table 2).

To optimize laboratory methods for obtaining high‐quality of HMW‐DNA from marine samples, we then tested silica‐based approaches that are reported ideal for high‐throughput DNA yield, especially from small tissue samples (Street et al., 2020). Additionally, liquid nitrogen homogenization has been substituted by mechanical homogenization of the target tissues in the corresponding lysis buffer.

High molecular weight genomic DNA extraction from marine invertebrates has been previously reported upon the addition of cetyltrimethylammonium bromide (CTAB) and polyvinylpyrrolidone (PVP) in the extraction buffer (Lin et al., 2016). These chemicals are known to bind to polysaccharides and polyphenolics and are commonly used in DNA extraction protocols for plants (Jobes et al., 1995). Consequently, we investigated the yield of the DNeasy Plant extraction kit. With this kit, high DNA concentration was observed, however, low purity was noted with noticeable smearing after gel electrophoresis. The reason of the low purity might be due to the missing protease digestion step resulting in a potential co‐precipitation of proteins during the elution as evident by the low A260/280 index (Chakraborty et al., 2020). We modified the protocol by including an extra Proteinase K step. The recorded purity index slightly improved but remained out of the recommended conditions for ONT.

When we assessed the DNA extraction by the tissue and stool protocols, the quantity and purity yield were higher than observed by the previously investigated protocols. It seems that the mechanical homogenization and the protease digestion are crucial steps to a higher DNA yield (Ramakrishnan et al., 2017). The Qiagen Tissues kit protocol uses two lysis buffers: ATL buffer containing the sodium dodecyl sulfate SDS that acts as a detergent and aids in cell lysis. It disrupts non‐covalent bonds in proteins to denature and unfold them; and the AL buffer that contains the chaotropic agent guanidinium chloride. The latter component promotes the lysis and also inactivates nucleases and promotes nucleic acid binding to silica membrane. Comparing the two kits, the Stool DNA extraction kit yielded higher DNA concentration. Stool samples are known to harbor a lot of inhibitory compounds that hinders downstream molecular analysis, so the extraction kit usually includes specific buffers to remove any potential inhibitors. InhibitEX Tablets are included in the kit, and they are recommended after the digestion step in order to bind to any potential inhibitory metabolites to clear them out prior to the genomic DNA extraction. The addition of these inhibitors’ removal could explain the higher DNA yield. While a distinct band of genomic DNA was detected on the agarose gel for the stool kit DNA extract, some nucleic degradation was observed. This observation was not surprising as according to the kit, the size of the DNA yield from Qiagen silica‐based column extraction kits should range from 100 bp to 30 kb. This degradation could be due to the harshness of the method, including several vortex steps and centrifugations at high speed, resulting thus in DNA shearing.

As the purity and quality of the DNA extracted by all commonly used protocols did not fulfill the requirements for ONT sequencing, we used an optimized combination of these protocols. Our optimized protocol (Figure 3) included the addition of some critical steps related to tissue maceration/inhibitors removal and the adjustments of proteinase K incubation as well as the standard extraction techniques (Pinto et al., 2000). Moreover, the established protocol includes initial washing steps with PBS buffer to remove any potential impurity and/or any excess buffer retained with the sample followed by low speed (4000 × *g*) centrifugation steps (PBS wash, debris, and inhibitors removal) to minimize DNA damage. The addition of these steps in combination with the Genomic Tips kit improved the quality of the DNA that, subsequently, was successfully sequenced on ONT platform.

The efficiency of the protocol was evaluated based on the output when handling fresh tissue, frozen tissue, and tissue stored in ethanol. Initially the investigated species were handed over from the marine experts at the Environmental Science Center where potentially new species are deposited and stored in ethanol. When sequencing issues were faced with the different DNA extracts by the commonly used chemical, protocols, and kits, the conservation method of the specimen was questioned as we suspected that it may have interfered with the quality of DNA. Accordingly, new fresh samples were requested from our marine collaborator (Co‐author: Dr. Bruno W. Giraldes), and thus the subsequent protocols, optimization steps, and the final established protocol were based on fresh tissues. However, due to sampling seasonality, resampling of other organisms was not possible. The optimized protocol was tested on the available species with different viscosity content: ethanol conserved jellyfish specimen (*Chrysaora* sp.) and frozen conserved shrimp specimen (*Palaemon* sp.). The sequencing output was not affected by the conservation mode of the sample as pore blockage was no longer observed during the runs and data acquisition was high.

In conclusion, we presented an extraction protocol for different marine species optimized to provide high‐quality and increased yields of HMW‐DNA for whole genome sequencing by ONT technology. Our work highlights the remarkable effects of some adjustments and additions to the Genomic Tips protocol that helped to achieve a pure and high molecular weight DNA. While the extracted DNA was tested on ONT platform and considering the improvements observed, we believe that this protocol could be applied to extract HMW‐DNA that is suitable for other molecular analysis or to be sequenced on other platforms.

## CONFLICT OF INTEREST

All authors declare no competing interests.

## AUTHOR CONTRIBUTIONS

**Sonia Boughattas:** Formal analysis (lead); Investigation (lead); Methodology (lead); Supervision (equal); Validation (equal); Writing – original draft (lead). **Dana Albatesh:** Formal analysis (supporting); Methodology (equal). **Albandari Al‐KHater:** Formal analysis (supporting); Methodology (supporting). **Bruno W. Giraldes:** Resources (lead). **Asma A. Althani:** Funding acquisition (equal); Project administration (equal). **Fatiha M. Benslimane:** Conceptualization (lead); Funding acquisition (lead); Project administration (equal); Supervision (equal); Validation (equal); Writing – review & editing (equal).

## Data Availability

The manuscript is a protocol paper with detailed methodology steps within the article text. So, no extra data are generated to be deposited.
